# Intestinal Anti-tissue Transglutaminase2 Autoantibodies: Pathogenic and Clinical Implications for Celiac Disease

**DOI:** 10.3389/fnut.2020.00073

**Published:** 2020-05-29

**Authors:** Mariantonia Maglio, Riccardo Troncone

**Affiliations:** Department of Medical Translational Sciences and European Laboratory for the Investigation of Food-Induced Diseases, University Federico II, Naples, Italy

**Keywords:** intestinal anti-TG2 antibodies, autoimmunity, gluten, celiac disease, intestinal production of anti-TG2

## Abstract

Celiac disease (CD) is a systemic disease that primarily affects the small intestine. The presence of anti-tissue transglutaminase 2 (anti-TG2) antibodies in the serum, as well as the presence of autoimmune phenomena, account for the inclusion of CD among autoimmune diseases. Anti-TG2 autoantibodies are produced at intestinal level, where they are deposited even before they appear in circulation. The pathogenic events that lead to their production are still not completely defined, but a central role seems to be played by gliadin-specific T cells. Interestingly, limited somatic mutations have been observed in VH and VL genes in TG2-specific plasma cells, another important aspect being the biased use of a heavy chain encoded by the VH5 gene. Conflicting data have been produced over the years on the effect of anti-TG2 antibodies on TG2 function. Although the presence of anti-TG2 antibodies in serum is considered a hallmark of CD and relevant from a clinical viewpoint, the role of these autoantibodies in the development of the celiac lesion remains to be defined. In the years, different technical approaches have been implemented to detect and measure intestinal CD-associated autoantibody production. Two aspects can make intestinal anti-TG2 antibodies relevant: from a clinical viewpoint: the first is their proposed ability in potential coeliac patients to predict the development of a full-blown enteropathy; the second is their possible role in revealing a condition of reactivity to gluten in patients with no circulating CD-associated autoantibodies. In fact, the detection of CD-specific autoantibodies production in the intestine, in the absence of serum positivity for the same antibodies, could be suggestive of a very early condition of gluten reactivity; alternatively, it could be not specific for CD and merely attributable to intestinal inflammation. In conclusion, the role of mucosal anti-TG2 antibodies in pathogenesis of CD is unknown. Their presence, the modalities of their production, their gluten dependence render them a unique model to study autoimmunity.

## Introduction

Celiac disease (CD) is an immune-mediated systemic disorder elicited in genetically susceptible individuals by the ingestion of gluten contained in wheat and related prolamines in rye and barley. The disorder is characterized by a variable combination of clinical manifestations depending on dietary gluten exposure, the presence in the serum of CD-specific antibodies (anti-tissue transglutaminase and anti-endomysium antibodies), and different degree of enteropathy. CD patients show the presence of alleles of class II major histocompatibility complex DQA1^*^0501-DQB1^*^02 (HLA-DQ2 haplotype) and/or DQA1^*^0301-DQB1^*^0302 (HLA-DQ8 haplotype) (1). The prevalence of CD in European Countries varies considerably from 0.2 to 3% (2, 3). Comparable prevalence data have been reported also in South America and the USA (4, 5). CD is a common disorder in North Africa, Middle East Countries and India as well (6, 7). Taken together, such rates of prevalence establish celiac disease as one of the most common genetically-based chronic diseases.

The expression of autoimmune phenomena (8, 9), first of all the presence in the serum of anti-tissue transglutaminase 2 (anti-TG2) antibodies (10), and the strict association with autoimmune disorders suggested ESPGHAN to define CD as a systemic autoimmune disease (1).

In this short review we focus our attention on intestinal anti-TG2 antibodies discussing the mechanisms of their production, their possible role in the pathogenesis of the disease and the clinical implications of their detection.

## T Cells in CD

**Figure 1:**
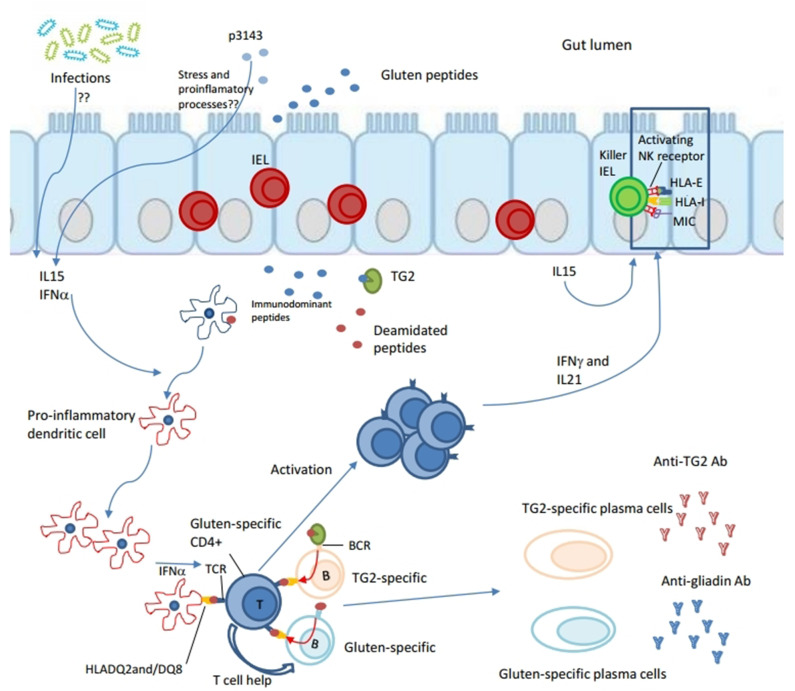
Both innate and adaptive immune responses are induced in the pathogenesis of celiac disease. Gliadin peptides resulting from the partial degradation of gluten in the intestinal lumen cross the epithelial barrier through the transepithelial way or passively by paracellular flux gaining access to lamina propria. Some gliadin peptides, such as p31–43, are thought to induce epithelial stress and inflammation. Other, the immunodominant ones such as the 33-mer, are deamidated by tissue transglutaminase 2 (TG2), resulting in higher affinity for HLA-DQ2 or DQ8 molecules. Deamidated gliadin-peptides are taken up by antigen presenting cells (APCs), such as pro-inflammatory dendritic cells, which promote activation of gluten-specific CD4+ T cell responses in *lamina propria*. Because of an already inflamed environment (by virus, gliadin? eliciting IL15 and type 1 interferons response), gluten-specific CD4+ T cells express a Th1 phenotype dominated by interferon (IFN)-γ and IL-21. Furthermore, gluten-specific T cells are thought to give help to both TG2-specific and gluten-specific B cells to differentiate into secreting anti-TG2 and anti-gliadin antibodies plasma cells, respectively. In particular, B cell recognizes its antigen (TG2-gliadin complex or gliadin peptides) by BCR, internalizes it, processes and presents gliadin in the context of HLA-DQ2 or DQ8 molecules to gliadin-specific CD4+ T cell. Because of their interaction both T cell and B cell would be activated producing pro-inflammatory cytokines, the first one, and differentiating in secreting plasma cell, the second. Other cytokines, such as IL15 and INF α are thought to be produced by stressed intestinal epithelial cells and/or dendritic cells. They are involved into innate immune responses. IL15 seems to have a central role in the recruitment of intraepithelial lymphocytes (IELs) and in their licensing to become pathogenic killer cells. In fact, IL15 seems involved in the expression of activating natural killer (NK) receptors CD94 and NKG2D, as well as in epithelial expression of stress molecules such as HLA-E and MIC. Finally, a complex remodeling of small intestinal mucosa takes place downstream of T-cell activation that leads to the classical “flat mucosa” of celiac disease.

The current concept of CD pathogenesis is that it involves components of both innate and adaptive immunity. Gluten is highly resistant to proteolytic degradation by mammalian and microbial intraluminal enzymes for its content of repetitive glutamine- and proline-rich sequences (11, 12). The incomplete degradation results in the persistence of gluten-derived gliadin peptides which access the *lamina propria*, either actively through the transepithelial way or passively by paracellular flux through damaged epithelial barrier (13, 14), inducing the activation of the mucosal immune system. It is still unclear if there is a primary defect of the barrier. The role played by some gliadin peptides, such as p31–43, is unclear too. They are thought to induce epithelial stress and proinflammatory events, which pave the way to the activation of the adaptive immune response (15–17). Other gliadin peptides, the immunodominant ones such as the 33-mer, activate in *lamina propria* gluten-specific CD4+ T cell responses presented by HLA-DQ2 or DQ8 molecules (18, 19). A key role in this process is played by tissue transglutaminase 2 (TG2) (20). The enzyme converts particular glutamine residues in gluten peptides to glutamic acid during a deamidation reaction. This results in higher affinity of these gliadin peptides for HLA-DQ2 or DQ8, thereby promoting the activation of T cells (21–23). Once activated, gluten-specific CD4+ T cells produce a pattern of pro-inflammatory cytokines dominated by interferon (IFN)-γ (Th1 skewed) and IL-21 (24, 25). Other cytokines, expression of the innate immune response, are also overproduced in the CD mucosa, such as IL15, IL18 and type 1 interferons. They are thought to be produced by stressed intestinal epithelial cells and/or dendritic cells (26–28). The mechanisms responsible of their recruitment are still unknown, but it is commonly accepted that cytotoxic intraepithelial lymphocytes are the key effector T cells mediating villous atrophy in celiac disease (29). They require complementary signals generated by adaptive anti-gluten immunity and epithelial stress to become pathogenic licensed killer cells (30). For the T lymphocytes intraepithelial recruitment, a key role seems to be carried out by IL-15 implicated in the expression of activating natural killer receptors CD94 (31) and NKG2D (32, 33), as well as in the expression of stress molecules at epithelial level (16). A complex remodeling of the mucosa takes place downstream of T-cell activation which leads to the classical “flat mucosa” of celiac disease. This process involve metalloproteinases (34) and growth factors (35), (Figure 1).

## Tissue Transglutaminase 2 (TG2) as Autoantigen

The first autoantibodies reported in CD were reticulin antibodies (36, 37) directed vs. collagen fibers (36, 38, 39). Subsequently, antibodies against endomysium, connective tissue that covers smooth muscle fibers, were identified (40). Anti-reticulin and anti-endomysium antibodies recognize in fact tissue transglutaminase 2 as proved by Dieterich et al. (10). TG2, the main autoantigen in CD, is a calcium dependent ubiquitous enzyme which exerts various functions. This enzyme has a wide distribution being localized in intracellular compartments and being released from cells in extracellular matrix during inflammation or as a consequence of tissue damage; moreover in extracellular space it is rapidly inactivated by oxidation (41). TG2 is involved in cellular processes of proliferation, of differentiation and apoptosis (42), of recycling endosomes (43), of cell adhesion and fibronectin assembly (44).

TG2 is believed to have at least two crucial roles in celiac disease: as a deamidating enzyme, that can enhance the immunostimulatory effect of gluten, and as a target autoantigen in the immune response. TG2 catalyzes post-translational modification of proteins forming isopeptide bonds; it catalyzes crosslinking between a glutamine residue (glutamine donor) and a lysine residue (glutamine acceptor) of a protein, resulting in the formation of an ε-(γ-glutamyl)–lysine (isopeptidyl) bond, process named transamidation (45). At a relatively low pH can also hydrolyse peptide-bound glutamine to glutamic acid introducing negatively charged residues, processed termed deamidation (45). This process generates negatively charged T cell epitopes in gluten peptides rising their bound affinity to CD-associated HLA molecules (21, 22). As known, the gliadin peptides are excellent substrates for this enzyme as up to 36% of their glutamine residues are accessible to deamidation by TG2 (46). Deamidation activity is believed to be a key step in the pathogenesis of celiac disease; it is a Ca++ dependent process, while it is inhibited when GDP binds to protein (47).

## Tissue Transglutaminase2 Antibodies: Mechanisms of Production

The adaptive immune response in celiac disease is characterized by the activation of gluten-specific CD4+ T cells in small intestinal mucosa. In the process leading to the antibody responses to both wheat gliadin and TG2 in the celiac small intestine, gluten-specific CD4+ T cells have a central role. In fact, no TG2 specific T cells have been identified. As known, production of anti-TG2 antibodies can develop only in HLA-DQ2 (48, 49) or HLA-DQ8 individuals (50) and recedes when gluten is excluded from the diet (51, 52). This observation led Sollid et al. (53) to formulate the so-called hapten-carrier hypothesis as a mechanism to explain how TG2-specific B cells get help from gliadin-specific CD4+ T cells to differentiate into IgA and IgG anti-TG2 plasma cells. B cells specific for TG2 recognize their antigens (TG2–gliadin complexes) via surface B cell receptors (BCRs), internalize them and present the processed gluten peptides to gluten-specific CD4+ T cells. Upon the interaction of HLA- DQ2 or HLA- DQ8, gliadin peptides and distinct T cell receptors (TCRs), both the T cells and the B cells would be activated. Once activated, gluten-specific CD4+ T cells start secreting inflammatory cytokines, thereby creating an inflamed environment in the small intestinal lamina propria and, on the other hand, the activated B cells can differentiate into plasma cells that secrete IgA and IgG antibodies against TG2 and deamidated gluten peptides. In this system, complexes formation between gliadin and TG2 makes gliadin a carrier protein for TG2. When gliadin is removed from the diet, T cell help for anti-TG2 specific B cells cease and the serum titers of anti-TG2 antibodies decline.

## Site of Production

The presence of anti-TG2 antibodies in serum is a hallmark of CD, being a very sensitive and specific marker to be used as diagnostic tool (54) and in the follow-up of treated patients (55). For years, it has been thought that antibody response in celiac disease originates in the intestinal mucosa. The first experimental evidence came from organ culture studies in which it was observed anti-endomysium antibody production by intestinal fragments after gliadin treatment (56). Conclusive in this regard are the studies by Marzari et al. (57) based on antibody libraries from the gut. They showed that the anti-TG2 autoantibodies are primarily synthesized by specific activated B lymphocytes in small intestinal mucosa of celiac disease patients, and their presence in the serum is probably attributable to the spillover from this source into the blood compartment. Furthermore, this study (57) showed that the heavy chain variable regions of these autoantibodies are primarily derived from the IGHV5-51 gene from the VH5 antibody variable gene family, indicating a preferential usage of this gene in the gluten-dependent autoimmune response to TG2 as subsequently confirmed by Di Niro et al. (58) using single-cell PCR on intestinal B cells.

The abundant local antibody production is confirmed by the observation that TG2 specific antibodies target the antigen in the extracellular matrix and the endothelium of small bowel vessels. A positive correlation between the percentage of TG2 specific plasma cells and serum levels and the intensity of mucosal IgA deposits was observed (59).

## Plasma Cells in the Celiac Mucosa

Approximately 1 and 10% of the plasma cells in small intestinal mucosa of celiac patients in the active phase of disease are specific for gliadin or TG2, respectively (58, 60). More recently, TG2 specific plasma cells have been found also in the lamina propria of 79% of potential CD patients before development of mucosal lesion (59). The density of TG2-specific plasma cells is considerably reduced in the celiac intestinal mucosa within 6 months since the start of gluten-free diet, but it remains elevated in comparison to no-CD patients (61). In fact, plasma cells specific for TG2 have been also found in subjects on a gluten free diet for several years (58). These cells could potentially live *in vivo* for months or years producing antibodies thanks to a favorable microenvironment. Di Niro et al. (58) with a cytometry-based method proved the intestinal production of anti-TG2 antibodies by plasma cells specific for TG2. They reported a very high density of plasma cells secreting TG2-specific IgA autoantibodies with limited somatic hypermutation in the damaged intestinal mucosa of active CD.

However, plasma cell function is not restricted to the production of antibodies. In fact, these cells appear to have a functional B cell receptor (BCR), together with expression of MHCII, as well as the co-stimulatory CD40, CD80, and CD86 molecules, which together may give them ability to activate T cells (62). In this context, recent evidence showed that both gluten-specific and TG2-specific plasma cells, but not dendritic cells and macrophages, were the most abundant cells presenting the immunodominant gluten peptide DQ2.5-glia-α1a in the lamina propria compartment of celiac mucosa (62). Plasma cells loading deamidated gliadin peptides will expand gliadin specific T cell clones. The amplification will provide further help to gliadin specific and TG2-specific B cells. The role played in the amplification of the T cell response renders plasma cells and B cells a target for future therapeutic strategies, even if antibodies do not seem to exert a direct pathogenic effect.

## Preferential Epitope Recognition and Usage of VH Genes

Another consequence of the T-B collaboration on the production of anti-TG2 antibodies is the definition of the enzyme epitopes. The autoantibody response against the TG2 in celiac disease patients has been investigated by generating recombinant antibodies from single gut plasma cells reactive with discrete antigen domains (58) and by undertaking proteomic analysis of anti-TG2 serum antibodies (63). Studying a panel of anti-TG2 antibodies produced by single plasma cells from damaged duodenal mucosa of active CD patients was clear that majority of them recognized epitopes in the N-terminal domain of TG2 (64). These antibodies did not interfere with enzymatic activity of TG2 (58) and did not impair antigen presentation to T cells, but it was not the same for the antibodies binding to C-terminal domain of TG2 (65). As proved, antibodies recognizing C-terminal epitopes interfered with TG2 cross-linking activity, and B cells specific for C-terminal epitopes were inefficient at taking up TG2-gluten complexes for presentation to gluten-specific T cells (65).

The intestinal autoimmune response to TG2 is characterized by an extensive switch to IgA occurring in the majority of plasma cells specific for TG2. Interestingly limited somatic mutations have been observed and this has led to the suggestion that their production takes place at extrafollicular sites more than at germinal centers (58). Another important aspect is the biased use of a heavy chain encoded by the VH5 gene. The reason for the preferential expansion of VH5 in the CD repertoire is not known. B cells expressing the IgH variable gene segment IgH V 5-51 have their B cell receptor (BCR) crosslinked to gliadin peptides by TG2 (66). This could account for the overrepresentation of IGHV 5-51 among TG2-specific plasma cells. This phenomenon could be specific for celiac disease. In fact, Maglio et al. (67) showed immunofluorescent detection of anti-TG2 autoantibodies mucosal deposits in 78% of type 1 diabetic children with serum positivity for the same antibodies. In these patients, the phage display analysis of intestinal antibodies libraries showed anti-TG2 antibodies made with a preferential use of the VH5 antibody gene family. At the same time, anti-TG2 antibodies belonging to other VH gene families, such as VH3 and VH1, were shown to be produced in the intestine of type 1 children with normal serum titers of the same antibodies. In the latter subjects high percentage was observed of phage antibodies belonging to VH3 gene family (67).

## Pathogenicity of TG2 Antibodies

The role of anti-TG2 and anti-gluten antibodies in the development of the celiac lesion remains to be defined. These antibodies are thought to increase the permeability of the epithelial barrier (68), allowing gliadin peptides to access the lamina propria and affecting epithelial cell biology (69, 70). Moreover, they may amplify the inflammatory immune response to gluten by increasing gluten uptake (71). Conflicting data have been produced over the years on the effect of anti-TG2 antibodies on TG2 function. Several research groups have investigated this topic with different results as to the degree of inhibition (72–76). Evidence have shown that IgA and IgG anti-TG2 autoantibodies from serum of CD patients as well as commercial monoclonal anti-TG2 perform a dose dependent and partial inhibitory effect on the transamidating activity of TG2 of gluten peptides. It has been shown that anti-TG2 antibodies interacting with the extra cellular bound TG2 induce relevant cytoskeleton changes with actin redistribution (70). Furthermore, interaction of anti-TG2 antibodies with extracellular TG2 triggers a rapid mobilization of calcium and the increased intracellular levels of calcium were able to activate intracellular TG2 (77).

Transglutaminase2 is part of a numerous enzyme family. The autoantibody responses targeting other members of this family are of great interest due to their association with specific clinical manifestations of celiac disease. In particular, anti-TG3 and anti-TG6 antibodies, which occur in the context of dermatitis herpetiformis and gluten ataxia, respectively, have been considered as potential contributors in the pathogenesis of these extra-intestinal manifestations (78, 79). Furthermore, anti-TG3 and anti-TG6 autoantibodies are the result of specific immune responses against TG3 and TG6, respectively, and are not the result of cross-reactivity to TG2 (64).

## Intestinal Anti-Tissue Transglutaminase 2 Antibodies: Tools for Detection

In the last 40 years different technical approaches have been used to detect and measure intestinal CD-associated autoantibody production: measurement of anti-reticulin antibodies in jejunal juice (80) or of anti-endomysium antibodies (EMA) and anti-TG2 IgA antibodies in feces (81), search of the same antibodies in cultured biopsy supernatants (56, 82–85) or detection of deposited anti-TG2 antibodies in duodenal fragments (86, 87), or of plasma cells secreting them (58) or, lastly, expression in intestinal phage libraries of RNA coding for the celiac autoantibodies (57), (Table 1).

**Table 1:** Tools employed to detect intestinal production of anti-TG2 autoantibodies.

**Tool**	**Technique**	**Strengths**	**Weaknesses**	**References**
Detection of anti-reticulin antibodies in jejunal juice	Indirect immunofluorescence on frozen sections of kidney rat		– Low sensitivity – Difficulty in getting the samples – Requires frozen samples of kidney rat and highly experienced operators – Unreliable as diagnostic test	(80)
Detection of EMA in fecal supernatants	Indirect immunofluorescence on frozen sections of monkey esophagus	– High sensitivity – Easy to get fecal samples	– Requires frozen monkey esophagus samples and highly experienced operators – Culture system technology requires experienced operators	(81)
Measurement of EMA in supernatants from cultured biopsy experiments	Indirect immunofluorescence on frozen sections of monkey esophagus	High sensitivity	– Requires frozen monkey esophagus samples and highly experienced operators – Culture system technology requires experienced operators	(56, 82, 88, 89)
Measurement of anti-TG2 IgA antibodies in fecal supernatants	ELISA	– Easy to get fecal samples	Sensitivity lower than serum tests	(81)
Measurement of anti-TG2 IgA antibodies in supernatants from cultured biopsy experiments	ELISA	– High sensitivity and specificity – ELISA Test is not dependent on operator skill – Quantitative test	Culture system technology requires experienced operators and equipped laboratories	(83–85, 88, 90, 91)
Detection of mucosal IgA anti-TG2 deposits	Double immunofluorescence on frozen duodenal sections	– High sensitivity – High specificity	– Requires frozen duodenal sections, highly experienced operators and equipped laboratories – Semiquantitative test	(84–87, 89, 91–93, 98)
Detection of synthesized IgA anti-TG2 from intestinal B lymphocytes	Phage display libraries	– High sensitivity – High specificity	– Tested in small patient groups and by only one group of researchers – Highly complex test that requires experienced operators and equipped laboratories – It cannot be proposed for routine use	(57, 94)
Detection of anti-TG2-specific plasma cells	Double immunofluorescence on frozen duodenal sections	– High sensitivity – High specificity	Requires frozen duodenal sections, highly experienced operators and equipped laboratories	(58, 59, 61)
Sorting TG2-specific plasma cells	Flow cytometry		Requires freshly picked duodenal samples, highly experienced operators and equipped laboratories	(58, 61, 64, 65)

*Anti-TG2, anti-tissue transglutaminase2 antibodies; EMA, anti-endomysium antibodies*.

The first evidence of antibody production in the celiac intestine dates back to the seventies, Mawhinney and Love (80), have demonstrated, recurring to indirect immunofluorescence on frozen sections of kidney rat, the presence of anti-reticulin antibodies in the jejunal juice of CD patients suggesting this feature as a result of stimulation of the secretory immune system. Afterwards, detection of EMA and anti-TG2 IgA antibodies in fecal supernatants, by indirect immunofluorescence analysis on cryostat sections of monkey esophagus and by ELISA test, respectively, represented one of the first proofs that intestinal mucosa is a site of celiac auto-antibody production (81). The authors proposed to recur to this measurement, together with the histological examination of multiple biopsies, as a useful diagnostic tool of CD in mucosae characterized by a patchy lesion; however, at a later time it was determined to be unreliable as a diagnostic test with a sensitivity lower than serum tests (95).

Further evidence in favor of the hypothesis of the intestinal production of the celiac-specific autoantibodies came from *in vitro* cultured small intestinal biopsy experiments. The search for EMA and/or anti-TG2 antibodies in supernatants collected after 24 h of CD small intestine culture (56, 82–85), recurring to indirect immunofluorescence and ELISA tests, respectively, revealed very high sensitivity and specificity. In the pediatric population ELISA test, that is objective and not influenced by the operator ability, showed intestinal production of anti-TG2 autoantibodies in 100% of CD patients in the active phase of disease with a specificity of 93% (84, 91). In potential celiac disease, when there are circulating CD-specific autoantibodies, but no signs of morphological mucosal injury, this assay proved that in 96% of (84) or in 100% of cases (89) CD intestinal mucosa produced and secreted autoantibodies. It was proposed in particular to improve the accuracy of CD diagnosis in subjects with mild enteropathy (84, 88) and in patients negative for serum antibodies (88–90). Moreover, while the measurement of secreted anti-TG2 in culture supernatants shows high sensitivity and specificity, currently organ cultures are used for only research purposes; it requires expertise and advanced techniques, but might be useful in special situations when other diagnostic methods are inconclusive (88, 89).

**Figure 2:**
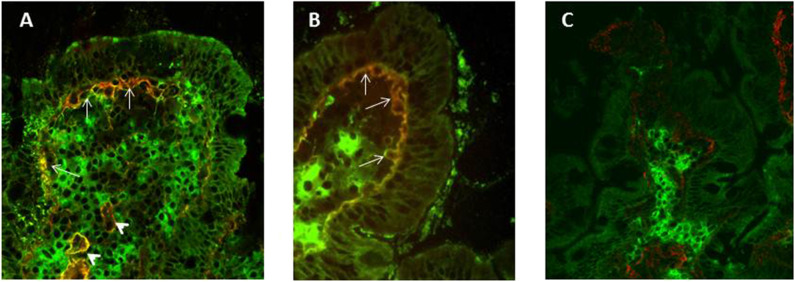
Double Immunofluorescence to detect mucosal deposits of immunoglobulin (Ig)A anti-tissue transglutaminase 2 (TG2). Duodenal mucosa sections from an active CD **(A)** and a potential CD **(B)** patient show bundles of IgA anti-TG2 antibody deposits in yellow, which are located under surface epithelium (white arrows) and around blood vessels (white arrowheads). IgAs secreted by plasma cells are detected in green **(A–C)**, TG2 with a sub-epithelial localization is shown in red **(C)**. In panel **(C)**, a duodenal section from a no-CD subject does not show mucosal deposits of anti-TG2 antibodies.

Using double immunofluorescence and frozen sections of duodenal mucosa, Korponay-Szabò et al. (86) highlighted thick bundles of mucosal IgA anti-TG2 deposits, secreted at intestinal level and deposited under surface epithelium and around crypts small intestinal mucosa of CD patients. This assay has been validated in several pathology laboratories for a diagnostic use, showing a sensitivity ranging from 73 to 100% (84, 87, 92, 93, 98) and a specificity ranging from 82 to 100% (87, 91, 92, 98). In potential CD patients deposits of IgA anti-TG2 are found less often (84, 87, 96) in about 68% of cases. The assay, while having high sensitivity and specificity, presents some limitations: requires frozen intestinal samples, equipped laboratory and highly experienced operators, (Figure 2). Tosco et al. (84) comparing the diagnostic efficiency of the detection of intestinal deposits of anti-TG2 antibodies and their measurement in biopsy culture supernatants in patients with potential CD, showed a higher diagnostic sensitivity and specificity for the measurement of secreted anti-TG2 antibody into culture supernatants than for immunofluorescence detection of mucosal deposits of the same autoantibodies.

Finally, by means of phage display libraries, Marzari et al. (57) showed that anti-TG2 IgA antibodies are synthesized primarily by specific B lymphocytes in the CD small intestinal mucosa and that in antibodies from celiac patients there is a preferential use of heavy chain variable regions belonging to the VH5 gene family. The phage display libraries technique seems to be more sensitive than other assays, such as detection of mucosal deposits of anti-TG2 antibodies using double immunofluorescence assay, in identifying patients with gluten-dependent intestinal or extra-intestinal symptoms, such as relatives of CD patients who show the presence of normal intestinal mucosa and the absence of serum anti-TG2 antibodies (94). However, this technology has been tested in small groups of patients and only from one research group; further studies are needed for validation. At the same time, it must be emphasized that, because of its complexity, this test cannot be proposed for routine use.

More recently, Di Niro et al. (58) by double immunofluorescence and flow cytometry methods identified TG2-specific plasma cells in duodenal intestine of CD patients in the active phase of disease and characterized their antibody response. Recurring to double immunofluorescence on duodenal cryosections it has been possible to detect TG2 specific plasma cells in all stages of CD but not in controls (58, 59, 61).

## Clinical Implications

### Intestinal Anti-TG2 in Active CD

Many studies investigated production of anti-TG2 autoantibodies in the intestine of patients with overt CD at diagnosis, by the tools described above. In adults with untreated CD anti-TG2 autoantibodies deposited in the intestine were detected in 100% of cases (92, 97, 98). In pediatric population more variability was reported. Mucosal deposits were identified in 96 to 100% of untreated CD patients (86, 87, 89, 93, 99). Considering the subgroup of children younger than 2 years of age this percentage is reduced (73%) (93). In this age group the search for mucosal deposits of anti-TG2–IgA did not improve the diagnostic performance in comparison to serum detection, (Table 2).

**Table 2:** Clinical implications of IgA anti TG2 antibodies.

**Intestinal anti-TG2 antibodies**	**Serum anti-TG2 antibodies**	**Possible diagnosis**
Deposited in the intestine and/or released from biopsy fragments	Very high titers	Celiac disease (with villous atrophy)
Deposited in the intestine (both with a patchy distribution and a weak staining) and/or released from biopsy fragments	Titers above the cut-off, <10x normal values	• Celiac disease • Potential celiac disease (predictive of development of villous atrophy?)
Deposited in the intestine (both with a patchy distribution and a very weak staining) and/or released in small amounts from biopsy fragment	Titers below the cut-off	• Very early celiac disease? • Non-coeliac intestinal inflammation • Autoimmunity (e.g., Type 1 diabetes)

Evidence showed the presence of anti-TG2 autoantibodies deposited in the small intestine of patients with dermatitis herpetiformis (DH) (86), a common extraintestinal manifestation of CD. Mucosal deposits were detectable also in DH patients with normal duodenal morphology and negative for serum specific-CD autoantibodies (86, 100) as well as in DH patients on a gluten-free diet (100).

Another gluten-related disorder investigated for the presence of mucosal deposits in the intestine is gluten ataxia. Anti-TG2 autoantibodies were showed in the gut and brain of patients with gluten ataxia with or without an enteropathy; these mucosal deposits were similar to those detected in the intestine of celiac disease and dermatitis herpetiformis patients but were not detected in subjects with other causes of ataxia. This finding strengthens the idea that gluten ataxia is immune mediated and belongs to the spectrum of gluten related disorders (101).

### Intestinal Anti-TG2 in Potential CD

Anti-TG2 autoantibodies production in the intestine has been investigated also in potential CD, a condition characterized by presence of anti-TG2 in the serum, but normal mucosa architecture. Several studies performed in adult and pediatric populations found the presence of mucosal deposits of anti-TG2 ranging from 68 to 100% (87, 89, 92, 97, 99, 102, 103). Tosco et al. (96) speculated that in a group of potential CD patients followed-up with repeated biopsy the detection of mucosal deposits of autoantibodies in the first biopsy could predict the later development of a full-blown villous atrophy, (Table 2).

### Intestinal Anti-TG2 in Subjects With Selective IgA Deficiency

The presence of mucosal deposits of anti-TG2 antibodies was also investigated in patients with selective immunoglobulin (Ig)A deficiency (SIgAD), that is a condition frequently associated with celiac disease. A 10–20-fold increased risk of CD may be observed in subjects affected by SIgAD. For this reason, screening for CD is mandatory in SIgAD patients and is performed searching serum IgG anti-TG2 autoantibodies. At intestinal level lack of secretory-IgA is replaced by a compensatory increase in secretory-IgM (104). Borrelli et al. (105) investigated by immunofluorescence the presence of IgM anti-TG2 antibodies in the intestine of SIgAD patients. The authors showed IgM anti-TG2 antibody deposits being present only in CD active patients with SIgAD. However, intestinal IgM anti-TG deposits did not discriminate between SIgAD and potential CD with SIgAD. Therefore, they concluded that the finding of serum IgG CD-associated autoantibodies remains crucially important in the diagnosis of CD in SIgAD.

### Intestinal Anti-TG2 in Patients on GFD

The disappearance of serum anti-TG2 antibodies is observed progressively after the beginning of a GFD. It has been considered a useful tool to evaluate the compliance with the diet, although it is clear that it is not able to reveal minor transgressions. It is also not yet established if it is helpful to assess complete histological recovery (106). Several studies were performed in CD patients on a gluten free diet to investigate the effect of the diet on anti-TG2 antibodies intestinal production. Studies on the follow-up of CD patients on GFD (107, 108) showed significant decrease of circulating anti-TG2 antibodies during the first 12 months of the diet and their disappearance in the next 12 months. Moreover, the disappearance from serum of specific CD autoantibodies, following a strict GFD, does not mean the end of their intestinal production. Many studies, involving pediatric or adult patients, showed the presence of intestinal anti-TG2 deposits both after a short term (1 year) and long term (>2 years) GFD (85, 98, 109). After the disappearance from serum, anti-TG2 antibodies as deposits would disappear slowly from the intestine, and only after a long period of GFD small intestinal mucosa would cease to produce them. Even when the mucosal deposits were not detectable in GFD patients, their intestine continue to produce low amounts of these antibodies in culture supernatants as recently showed (85). Further evidence of intestinal anti-TG2 production, even after years of gluten free diet, came from flow cytometry cell sorting of TG2 specific plasma cells from duodenal samples of treated CD patients (58, 61). More recently, Risnes et al. (110) recurring to HLA-DQ:gluten tetramers technology proved the persistence for decades of gluten-specific T cells, which are thought to play a central role in antibody production process, in the intestine of treated CD. Interestingly, during a GFD the CD markers normalize in a sequential order: serum anti-TG2, morphology of small intestine, density of CD3+ intraepithelial lymphocytes and then mucosal deposits. The only marker that remained positive also very late is the high density of TCR-γδ+ intraepithelial lymphocytes (98).

### Intestinal Anti-TG2 in Subjects Seronegative: Gluten-related or False Positive?

Intestinal anti-TG2 antibodies may be present early, before they appear in the serum. Borrelli et al. (111), having available very early biopsies from at-risk infants, enrolled in the context of the European multicenter project Prevent Celiac Disease (PreventCD, www.preventcd.com) (112), investigated the appearance of intestinal anti-TG2 deposits and their predictive value for villous atrophy. The authors showed anti-TG2 antibodies deposited in duodenal mucosa of all subjects who had diagnosis of CD. However, mucosal IgA anti-TG2 deposits were also present before the mucosal damage and even before they appeared in serum.

Identifying intestinal production of anti-TG2 becomes important in support of a diagnosis of celiac disease in borderline cases, mainly in patients with absence of serum CD-associated autoantibodies (87, 89, 91, 113) which include relatives of CD patients that Not et al. have defined as affected by cryptic genetic gluten intolerance (94). The production of anti-TG2 antibodies was showed in the intestine of DQ2- or DQ8-positive relatives of CD patients even in the presence of a normal intestinal mucosa and without circulating anti-TG2 antibodies by detection of mucosal deposits (89, 91, 114) and by mucosal phage display antibody assay (94). These patients have been shown produce only at intestinal level IGVH5-51 anti-TG2 antibodies, in other words using the same VH genes found in overt celiac patients. Interestingly the presence of symptoms in these subjects responsive to the gluten free diet, placed them in the spectrum of gluten-related disorders.

Several investigators have shown that intestinal anti-TG2 IgA deposits were also detectable in subjects with diagnosis other than CD (87, 97, 98). The data were confirmed by measuring anti-TG2 antibodies spontaneously secreted into supernatants from organ culture of small intestinal biopsy and by the phage display technologies (91). Indeed, the construction of antibody libraries from biopsy samples showed a production of an anti-TG2 specific antibody repertoire dominated by the usage of VH5-51 gene segment and by the VH3 gene segment. The presence of intestinal anti-TG2 have been reported, in most of such cases associated with other autoimmune disorders such as type 1 diabetes (T1D). In all these conditions, the relation of intestinal anti-TG2 autoantibodies to CD is difficult to establish, the detection of mucosal deposits might be a very early sign of gluten sensitivity or simply a “false-positive” staining. It is possible that intestinal anti-TG2 is an expression of mucosa inflammation related to the autoimmunity state. As already mentioned, a high prevalence of anti-TG2 intestinal deposits has been reported in patients with T1D on a normal diet irrespective of the presence of serum anti-TG2 antibodies (67). However, only T1D patients with elevated serum levels of anti-TG2 antibodies showed the VH5-51 gene usage typical of the anti-TG2 antibodies produced in the intestine of CD patients, suggesting a gluten-dependent phenomenon. In serum anti-TG2 negative T1D patients, who produced and deposited intestinal anti-TG2 antibodies belonging to the VH-1 and VH3- gene families (67, 115), the relationship with dietary gluten is uncertain, (Table 2).

### Intestinal Anti-TG2 in Relation With Other Gluten-Related Bio-markers

Many evidences related the presence of mucosal anti-TG2 deposits with other CD markers. The density of TCR-γδ+ intraepithelial lymphocytes is considered highly sensitive and specific for CD (116). In fact, high density of this marker has been shown to be indicative of early developing CD (103). Mucosal deposits of anti-TG2 antibodies have been reported in children who were negative for serum CD-associated autoantibodies but with an increased number of TCR-γδ+ intraepithelial lymphocytes, with mucosal deposits of anti-TG2 antibodies (117). It remains to be established if these subjects belong to the spectrum of gluten related disorders.

## Conclusions

Anti-TG2 autoantibodies are a key feature of celiac disease; their intestinal production as well as the deposition in the mucosa, even before their appearance in serum, have been extensively proved. Intestinal anti-TG2 antibodies can be considered to be relevant from a clinical viewpoint for two reasons: the first is their supposed capability to predict the development of a full blown enteropathy in a early phase disease condition such as potential celiac disease; the second is their possible role in revealing a condition of gluten reactivity in patients with absence of circulating CD-associated autoantibodies. Alternatively, the detection of specific CD autoantibodies production in the intestine, in the absence of serum positivity for the same antibodies, could be not specific for celiac disease, but a phenomenon merely attributable to intestinal inflammation.

As previously mentioned, the pathogenic role of mucosal anti-TG2 antibodies in CD is unknown. However, their presence, the modalities of their production, their gluten dependence render them a unique model to study mechanisms of autoimmunity.

## Author Contributions

MM and RT contributed equally to the planning and drafting of the manuscript.

## Conflict of Interest

The author declares that the research was conducted in the absence of any commercial or financial relationships that could be construed as a potential conflict of interest. The handling Editor declared a past co-authorship with the authors RT.
